# Synergistic Defensive Function of Raphides and Protease through the Needle Effect

**DOI:** 10.1371/journal.pone.0091341

**Published:** 2014-03-12

**Authors:** Kotaro Konno, Takashi A. Inoue, Masatoshi Nakamura

**Affiliations:** 1 National Institute of Agrobiological Sciences, Tsukuba, Ibaraki, Japan; 2 National Institute of Agrobiological Sciences, Hokuto Campus, Hokuto, Yamanashi, Japan; Beijing Forestry University, China

## Abstract

Raphides, needle-shaped calcium oxalate crystals in tissues of many plants, have been thought to play defensive roles against herbivores without detailed bioassays for their defensive roles and modes of function using purified raphides. In order to examine the defensive roles and modes of function of raphides in a clear experimental system, we performed bioassays giving the larvae of the Eri silkmoth, *Samia ricini* (Saturniidae), leaves of their host plant, the castor oil plant, *Ricinus communis* (Euphorbiaceae), painted with the raphides purified from kiwifruits, *Actinidia deliciosa* (Actinidiaceae), in presence or absence of cysteine protease, which often coincide with raphides in plant tissues. Raphides alone or cysteine protease alone showed only weak defensive activities around experimental concentrations. However, when raphides and cysteine protease coexisted, they synergistically showed very strong growth-reducing activities, and the mortality of caterpillars was very high. In contrast, amorphous calcium oxalate did not show synergism with cysteine protease on defensive activities, indicating that the needle-shape of raphides is essential for the synergism. The present study provides the first clear experimental evidence for the synergism between raphides and other defensive factors. Further, the study suggests that “the needle effect”, which intensify the bioactivities of other bioactive factors by making holes to the barriers (cell membrane, cuticle, epithelium, the nuclear membrane, etc.) and facilitate the bioactive factors to go through them and reach the targets, is important in the defensive activities of raphides, and possibly in the allergy caused by raphides, and in the carcinogenic activities of other needle-shaped components including asbestos and plant derived silica needles.

## Introduction

**Figure 1:**
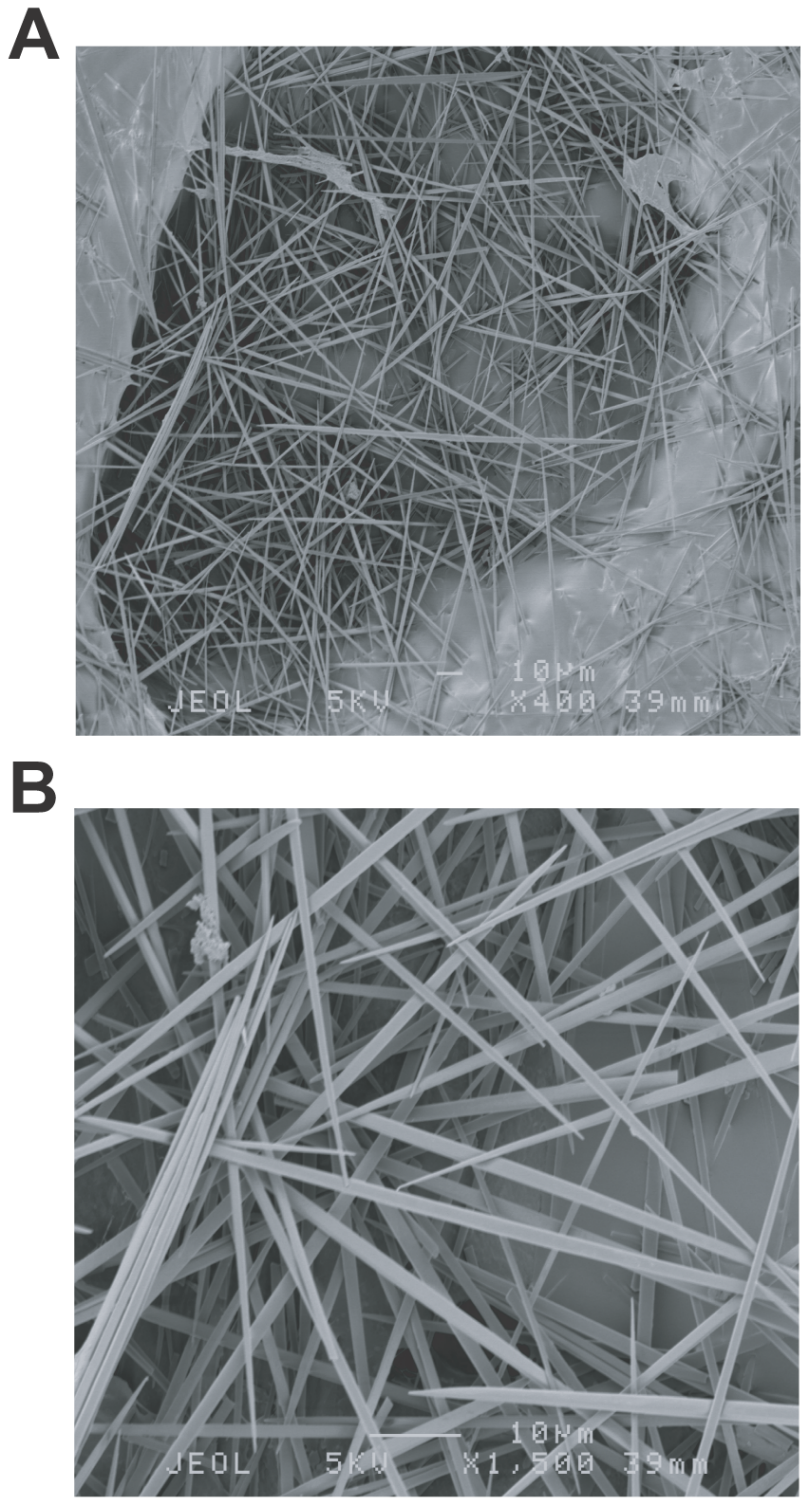
Raphides purified from kiwifruit. Raphides, needle-shaped calcium oxalate crystals, were collected from kiwifruit homogenate through heavy media separation using a dense CsCl solution. (A) The SEM image of purified raphides (x400). (B) Further magnification of the left part of the image (A) (x1,500). The white bars in photos indicate 100 μm (or 0.1 mm). Raphides purified from kiwifruit had sharp needle-like shapes with smooth surfaces, and did not have grooves and barb-like shapes observed in raphides of Araceae plants [12].

Raphides are sharp needle-shaped crystals of calcium oxalate (Figure 1) found in various tissues including leaves, roots, shoots, fruits, etc., of wide varieties of plant species, and are typically kept in highly specialized cell called idioblast [1], [2]. Raphides are commonly found in monocots families such as Araceae, Agavaceae, Orchidaceae, Smilacaceae, Discoreaceae, Bromeliaceae, Arecaceae, Commelinaceae, Musaceae and also in some dicot families such as Rubiaceae, Solanaceae, Actinidiaceae, Vitaceae, [2]–[6]. In spite of their wide distribution and conspicuous appearance, their primary roles have not been well explained and several hypothetical roles have been raised including calcium regulation, plant defense against herbivores, detoxification of aluminum, etc., which are not necessarily mutually exclusive [1]. Among these proposed roles, defensive roles of raphides against herbivores have several supporting observations, of which the oldest one dates back to a century ago when a German scientist Ernst Stahl first observed the phenomenon that snails avoided to eat plant materials containing raphides, but once the materials were treated with acid to dissolve calcium oxalate, snails consumed the treated plant material well [7], [8]. Other observations include observation that stinging, irritating and inflammatory activities of Araceae plant tissues (leaves and tubers) could be reproduced by collected raphides from plant tissues of the Araceae plants [9], [10], observation that all three herbivore species gazelle, moth larvae and snail fed only on tips of the leaves of a desert lily, *Pancratium sickenbergeri* (Amarylidaceae), where raphides were absent [11], and observation that raphides from some plants had a barb-like shape ideal to penetrate into through tissues of herbivores [12]. Above studies suggested that raphides have defensive roles against herbivores, however, detailed bioassays using purified raphides without other plant materials designed to estimate the defensive roles of raphides as plant defense mechanism and their detailed modes of actions have been scant. Further, when considering carefully, there are lot of controversial observations that need to be explained. Why raphides of some plant or some raphide-containing plants are very irritating and toxic (dieffenbachia) [10], while those from other plant species or other plants not (grape, kiwi, pineapple, tomato etc.). Why raphides of some giant taro and taro cultivars show strong acridity, while those of other cultivars show only weak acridity even though there are no significant differences in amounts and morphology of raphides between acrid and non-acrid cultivars [9]? The only reasonable explanation to this question we thought was that some other defensive factor or defensive substances are substantially involved in the defensive activity of raphides. Since raphides are needles, and they may possibly make holes to the body, tissues or cells of herbivores that facilitate defensive factors or toxins to enter body, tissues or cells, we thought synergism between raphides and other defensive factors is possible and reasonable. Although this possibility has been vaguely implied or discussed in some previous studies [12], [13], there have been no direct attempts to examine the hypothesis by clear-cut experiments. In correspondence with this hypothesis, raphides often coincide in same tissues with other defense-related proteins against herbivores. For example, in kiwifruit and pineapple, raphides coincide with cysteine proteases [2], [5], [14](namely actinidin and bromelain, respectively), which are recently found by us and other scientist to functions as potent defense protein against herbivorous insects [15], and in yam tubers (*Dioscorea*) raphides coincide with chitinase [2], [3], [16], which function as defense protein against insect herbivores [17].

Therefore, the purpose of the present study is first, to examine the defensive activity of raphides against herbivorous insects using purified raphides, and second, to assess by clear-cut experiments whether synergism exists between raphides and cysteine protease in terms of defensive activity against herbivorous insects, and we succeeded in detecting strong synergistic defensive function of raphides and cysteine protease.

## Results

### Synergistic defensive effects between raphides and cysteine protease

**Figure 2:**
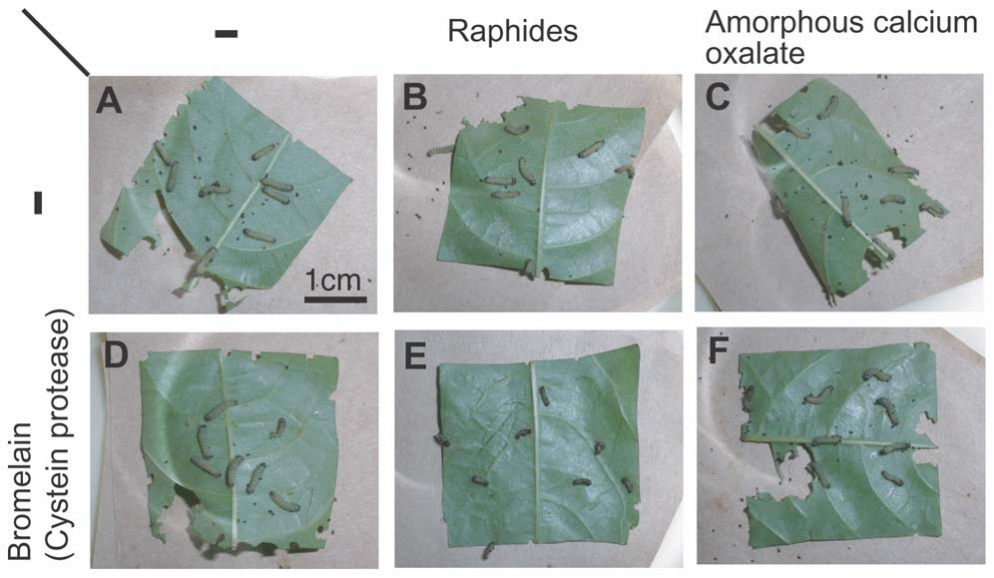
A bioassay on synergistic defensive effects between raphides and cysteine protease. Eight neonate larvae of the Eri silkmoth (*Samia ricini*, Saturniidae) (1.5 mg) were fed a leaf piece (3×3 cm pieces, ca. 0.1 g) of its host plant, the castor oil plant (*Ricinus communis*, Euphorbiaceae), painted either with calcium oxalate crystal (needle-shaped raphides purified from kiwifruits or commercially available amorphous crystals), with cysteine protease (bromelain) or with both for 1 day. (**A**) A control leaf piece of castor oil plant (painted only with CaCl2 and glycerol). (**B**) A leaf piece painted with raphides (42 μg/cm2 or 3.75 mg/g fresh leaf). (**C**) A leaf piece painted with amorphous calcium oxalate crystals (42 μg/cm2). (**D**) A leaf piece painted with bromelain (0.22 mg/cm^2^ or 5.76 unit/mg fresh leaf), a cysteine protease from pineapple stems. (**E**) A leaf piece painted with both bromelain (0.22 mg/cm^2^) and raphides (42 μg/cm2). (**F**) A leaf piece painted with both bromelain and amorphous calcium oxalate crystals (42 μg/cm2). Note that very strong defensive effects that resulted in high mortality were observed only when bromelain and raphides were fed together.

In order to assess the defensive activity of raphides against herbivorous insects, we fed the neonate larvae (1.5 mg) of the Eri silkmoth, *Samia ricini* (Saturniidae), with the leaves (3 cm×3 cm square leaf pieces, ca. 0.1 g) of the castor oil plant (*Ricinus communis*, host plant of the Eri silkworm) painted with raphides (0.375 mg, 42 μg/cm2, or 3.75 mg/g fresh leaf defined as 1 amount of raphides) collected and purified from kiwifruit (*Actinidia deliciosa* variety Hayward) (Figure 1), which was previously identified to consist of calcium oxalate by chemical analyses [5].The larvae fed the leaf pieces painted with raphides for 1 day (Figure 2B) showed significant but slight reduction of growth (4.28±1.06 mg) compared to the larvae fed control leaf pieces without raphides (5.17±1.28 mg) (Figure 2A), and no mortally was observed (Figure 2AFigure 2A,B; Table 1). The larvae fed the leaf pieces painted with bromelain, a cysteine protease from pineapple stems (0.22 mg/cm^2^ or 5.76 unit/mg fresh leaf, defined as 1 amount of bromelain) also showed significant but slight reduction of growth (3.52±1.44 mg) and some mortalities (25%) (Figure 2D (no mortality observed in this trial); Table 1). These result indicated that both raphides and bromelain have weak defensive effects in above experimental concentrations. Surprisingly, when larvae were fed the leaf pieces painted with both 1 amount of raphides (42 μg/cm2) and 1 amount of bromelain (0.22 mg/cm^2^) together, the larvae showed strong and significant reduction of growth (1.41±0.49 mg, eventually no growth), and very high mortality (86%) (Figure 2E (all died in this trial); Table 1). The dead bodies of the larvae are black and soft (Figure 2E), which symptom are typically seen when a large dose of cysteine proteases are fed to the larvae [15], and the toxic effects were very acute that the larvae start to shrink and stop feeding within 2 hours after the beginning of the bioassay. We further made bioassays in order to clarify whether the strong defensive activity observed when both raphides and bromelain were present are the result of synergistic effect of both factors or mere result of additive effect of both factors. When larvae were fed leaf pieces painted either with 2 amounts of raphides (83 μg/cm2) or 2 amounts of bromelain (0.44 mg/cm2), larvae still showed only a weak growth reduction (4.02±0.50 mg and 2.99±1.27 mg, respectively) and low mortality (0% and 8%, respectively), which is in a great contrast with the strong growth reduction (1.41±0.49 mg) and high mortality (86%) observed with the larvae fed leaf pieces painted with both 1 amount of raphides and 1 amount of bromelain together (Table 1). These results indicate that raphides and bromelain exert synergistic defensive effects.

**Table 1:** Synergistic defensive effects of raphides and cysteine protease.

	Larval mass (mg)	Mortality (%)	*n*
Control (Leaves of the castor oil plant painted only with CaCl_2_ and glycerol)	5.17±1.28^a^	0	29
+Raphides (42 μg/cm^2^ or 3.75 mg/g fresh leaf)	4.28±1.06^b^	0	28
+Raphides (83 μg/cm^2^ or 7.50 mg/g fresh leaf)	4.02±0.50^bc^	0	12
+Bromelain (0.22 mg/cm^2^ or 5.76 unit/mg fresh leaf)	3.52±1.44^bd^	25	28
+Bromelain (0.44 mg/cm^2^ or 11.52 unit/mg fresh leaf)	2.99±1.27^cd^	8	12
+Raphides (42 μg/cm^2^) + Bromelain (0.22 mg/cm^2^)	1.41±0.49^e^	86	28
+Amorphous calcium oxalate (42 μg/cm^2^)	2.92±0.53^cd^	0	16
+Amorphous calcium oxalate (42 μg/cm^2^) + Bromelain (0.22 mg/cm^2^)	2.83±0.83^d^	19	16

Neonate Eri-silkmoth larvae (1.5 mg) were fed leaves of their host plant (castor oil plant) together with raphides, bromelain or both for 1 day, and the larval mass (average ± SD) and mortality were measured. Values of larval mass not followed by the same letters are significantly different (*P*<0.01; Tukey's test for multiple comparisons). The definition of the unit for protease activity is based on casein digestion, and is described in Materials and Methods.

In order to examine whether raphides and bromelain show synergistic defensive activity in the low dose range, we performed another series of bioassays feeding larvae with leaf pieces painted with 1/8 amount of raphides (5.2 μg/cm^2^ or 0.469 mg/g fresh leaf) alone, 1/10 amount of bromelain (0.022 mg/cm^2^ or 0.576 unit/mg fresh leaf) alone, or both together for one day (Table 2). While neither larvae that were fed leaves that were painted with 1/8 amount of raphides alone (4.51±0.25 mg) nor those that were fed leaves painted with 1/10 amount of bromelain (4.70±0.37 mg) alone showed significant growth reduction compared to those that were fed control leaves (4.65±0.36 mg), the larvae that were fed leaves painted with both 1/8 amount of raphides and 1/10 amount of bromelain together showed moderate but significant growth reduction (3.85±0.52 mg) compared to the larvae under any of above three treatments. These results indicate that raphides and bromelain exert synergistic defensive effects even in the low dose range in which neither raphides nor bromelain alone shows defensive activity.

**Table 2:** Synergistic defensive effects of raphides and cysteine protease in the low dose range.

	Larval mass (mg)	Mortality (%)	*n*
Control (leaves of the castor oil plant painted only with CaCl_2_ and glycerol)	4.65±0.36^a^	0	24
+Raphides (5.2 μg/cm^2^ or 0.469 mg/g fresh leaf)	4.51±0.25^a^	0	24
+Bromelain (0.022 mg/cm^2^ or 0.576 unit/mg fresh leaf)	4.70±0.37^a^	0	24
+Raphides (5.2 μg/cm^2^) + Bromelain (0.022 mg/cm^2^)	3.85±0.52^b^	4	24

Neonate Eri-silkmoth larvae (1.5 mg) were fed leaves of their host plant (castor oil plant) together with raphides, bromelain or both for 1 day, and the larval mass (average ± SD) and mortality were measured. Values of larval mass not followed by the same letters are significantly different (*P*<0.01; Tukey's test for multiple comparisons). The definition of the unit for protease activity is based on casein digestion, and is described in Materials and Methods.

In order to relate the activity of protease and amount of raphides used in bioassays with those in natural conditions, we analyzed the protease activity of the kiwifruits. The protease activity of newly harvested kiwifruits (cultivar Hayward) analyzed using casein as substrate was 0.461±0.161 unit/mg fresh fruit, whereas previous studies [5], [18] suggested that the amounts of raphides in fruits and leaves of kiwifruit plant, which we calculated from amounts of insoluble oxalate in kiwifruit tissues reported in these studies, were 0.356 mg/g fresh fruit and 0.398 mg/g fresh leaf, respectively. (It is clear from the SEM images of uncontaminated raphides in Table 1 and from the purification method of raphides based simply on density that there are no structures other than raphides in kiwifruits that are composed of insoluble oxalate salts with large densities such as druses or amorphous calcium oxalate crystals, which then indicates that the amounts of insoluble calcium oxalate in kiwifruits in above studies well represent the amounts of raphides in kiwifruits.) These results indicated that the doses of protease and raphides used in above bioassays, especially the doses in the low dose range bioassay, well represent the activity of protease and the amount of raphides in natural conditions in kiwifruits.

### Importance of needle-like shape in the synergism

Synergistic defensive effects were not observed between amorphous calcium oxalate crystals and bromelain. When the larvae were fed leaf pieces painted with amorphous calcium oxalate crystals and bromelain, together, the larvae showed only a moderate growth reduction (2.83±0.83 mg) and a low mortality (19%) (Figure 2F; Table 1) that were not significantly different from those of the larvae fed amorphous calcium oxalate crystals alone (2.92±0.53 mg, 0%) (Figure 2C; Table 1) or bromelain alone (3.52±1.44 mg, 8%) (Figure 2D; Table 1). These results indicate needle-like shape of raphides is important for synergistic defensive effects of raphides and bromelain.

### Synergism between raphides and kiwifruit extract free of raphides

Similarly, synergistic defensive effects were observed between raphides and kiwifruit extract from which raphides were removed (Table 3). The larvae that were fed leaf pieces painted with raphides and kiwifruit extract together showed strong growth reduction or no growth (1.30±0.30 mg) and moderate mortality (31%), while the larvae that were fed leaf pieces painted with kiwifruit extract alone or with amorphous calcium oxalate crystals showed significantly weaker growth inhibition (2.38±0.23 mg and 2.94±1.06 mg, respectively) without mortalities (Table 3). These results suggested synergistic defensive effects also exists in kiwifruit, presumably between raphides and actinidin (actinidain), a cysteine protease in kiwifruit.

**Table 3:** Synergistic defensive effects of raphides and kiwifruit extract from which raphides were removed by centrifugation.

	Larval mass (mg)	Mortality (%)	*n*
Control (leaves of the castor oil plant painted only with CaCl_2_ and glycerol)	5.17±1.28^a^	0	29
+Kiwifruit extract (raphides removed) (4.4 μl/cm^2^)	2.38±0.23^b^	0	16
+Centrifuged kiwifruit extract (raphides removed) (4.4 μl/cm^2^) + Raphides (42 μg/cm^2^)	1.30±0.30^c^	31	16
+Kiwifruit extract (raphides removed)(4.4 μl/cm^2^) + Amorphous calcium oxalate (42 μg/cm^2^)	2.94±1.06^b^	0	16

Neonate Eri-silkmoth larvae (1.5 mg) were fed castor oil plant leaves painted with kiwifruit homogenate from which raphides were removed by centrifugation, alone, with raphides, or with amorphous calcium oxalate for 1 day, and the larval mass (average ± SD) and mortality were measured. Values of larval mass not followed by the same letters are significantly different (*P*<0.01; Tukey's test for multiple comparisons).

## Discussion

The present study with direct bioassays using purified raphides and pure cysteine protease clearly demonstrated for the first time experimentally that raphides exert strong plant defense effects against insect herbivores by synergistically intensifying other defensive factors, including cysteine proteases. Our study also showed that the needle shape of raphides, but not calcium oxalate as a material, is important for the synergistic effects. Further, the symptoms of the larvae fed leaf-pieces that were painted with raphides and bromelain were typically seen in the larvae that were fed cysteine protease in large amounts (i.e. the bodies of the deceased individuals were black and soft due to the digestion by protease) [15]. Therefore, it is reasonable to suppose as followings: raphides made holes somewhere in the body barriers, and thereby facilitating the entry of cysteine protease into the body and its digestion of the body from within. This scenario could be generalized as a “needle effect”, in which sharp needle-like structures intensify the bioactivity of other factors by making holes in physical barriers such as the epithelium, cuticle, cell membrane, nucleus membrane, etc., and helping the bioactive factors to reach their targets inside these barriers.

The concept of synergism between raphides and other defensive factors would be important for understanding the mode of the functions of raphides and for answering questions (or solving paradoxes) as to why some plant species or cultivars containing raphides have strong irritating effects while others containing raphides do not [9], [10], and why raphides sometimes coincide with other defensive factors (e.g., cysteine proteases in pineapple and kiwifruit) [2], [5], [14]. In short, one of the most important implications of the present study is that some defensive or bioactive factors other that raphides may be involved in the defensive activity and other bioactivities of raphides. Therefore, the discovery of synergistic effects between raphides and other defensive factors would be the first significant progress after the study by Ernst Stahl a century ago [7], [8], in understanding the defensive function of raphides against herbivores. In future, however, it is necessary to further confirm the generality of the synergism between raphides and defensive factors against herbivores, by using defensive factors other than cysteine protease, by using herbivores other than the Eri silkworms, and by testing the effects in various doses and concentrations of raphides and defensive factors and by comparing the doses and concentrations in bioassays with those in the natural conditions in plant tissues. Also, it is necessary to further substantiate and confirm the processes involved in the “needle effect”, such as by directly observing the holes made by raphides and identifying the locations of the holes in the body, and by directly detecting the increase of defensive factors that enter the body.

Synergisms between defensive factors, however, are not rare in plant defense systems. One type of synergisms is found in “precursor of toxin-activating enzyme-systems”, such as in cases of cyanogenic glucosides [19] and iridoid glucosides [20] releasing cyanate and glutaraldehyde-like aglyocone, respectively, both of which are harmful to herbivores, by the activity of β-glucosidase. Glucosinolate-myrosinase system [21] and phenolics-polyphenol oxidase system [22], that produce harmful isothiocyanate and quinones, respectively, are also such examples of synergism. The second type of synergisms in plant defense systems is “toxin-detoxification enzyme inhibitor-systems”. For example, linear furanocoumarins (toxin) and angular furanocoumarin, which function as inhibitor of P450 enzyme (detoxifying enzyme) that detoxify linear furanocoumarin, coexist in the same plant [23]. The third type of synergisms is “toxin-deliverer-systems”, in which the delivery of defense toxins to the target herbivores, tissues, or molecules is facilitated by physical structures (or chemicals or enzymes). Canalicular defenses such as plant latex, oil ducts and exuding phloem sap of cucurbit plants are included in this type, because these canalicular structures help the toxins rush to and exude at the point of damage by herbivores and help toxin reach target herbivores [24]–[26]. Synergism of raphides and cysteine protease reported in the present study is suitably categorized as the third type because raphides help protease enter the body and reach target (protein inside the bodies).

With our present study, the concept of raphide-borne defense become more firmly established and deeply understood. Our present study, together with the fact that raphides-borne defenses are very widely but sporadically found in the plant kingdom and that they are likely to have evolved convergently in many plant taxa independently, indicates that raphide-borne defenses can be regarded as a defense syndrome of plants against herbivores. Therefore, studying raphide-borne defense systems in may plant taxa will render a lot of empirical evidence important in understanding the evolutionary patterns (and their driving force) of plant-herbivore interaction as in the case of latex-borne plant defense systems, a well-studied plant defense syndrome [27].

The concept of “needle effects” could be important one to understanding various biological effects that raphides are involved, including allergenic effects and acridity, because for both allergenic effects and acridity, it is necessary for the allergenic and acrid factors to go into the body somehow, and the needle effects of raphides will greatly facilitate these processes. For example, a certain studies report that kiwifruit has allergenic effects and that actinidin, a cysteine protease in kiwifruit, plays important role in the allergenic effect without referring to raphides [28]. Meanwhile, other studies report that the raphides of kiwifruit as an irritant factor of kiwifruit without referring to protease [5]. It is very likely, however, that raphides and actinidin synergistically function in the allergenic or irritating activities of kiwifruit. Similarly, allergenic activity (dermatitis forming activity) reported from raphide-containing plants such as *Agave tequilana* [29], could be explained as synergism between raphides and allergenic protein in plants. The questions about controversial observations as to why raphides of some plants or some raphide-containing plants are very irritating and acrid (dieffenbachia) [10], while those from other plant species or other plants not (grape, kiwi, pineapple, tomato etc.), and as to why raphides of some giant taro and taro cultivars show strong acridity, while those of other cultivars show only weak acridity even though there are no significant differences in amounts and morphology of raphides between acrid and non-acrid cultivars [9], can be reasonably explained when we assume “needle effects” and existence of acrid factors that are synergistically enhanced by raphides.

Finally, the concept of “needle effects” could be an important one for clarifying and understanding the biological phenomena of other needle-like structures including the carcinogenic effects of needle-like structure such as asbestos [30], [31] and silica needles in plants [32], since needle-like crystals may facilitate weak carcinogens that cannot easily pass through barriers and therefore are not regarded as carcinogens under ordinary conditions, to go through barriers (cuticles, epithelia, cellular and nuclear membranes) and reach their targets (i.e. DNA in the nucleus), thereby raising the rates of carcinogenesis. The insolubility of asbestos and silica needles to water would result in the long accumulation of such “needle effects”, and thus a high total rate of carcinogenesis, while raphides consisting of calcium oxalate would be slightly soluble to water and have much shorter life spans, which could result in a smaller accumulation of the needle effects in terms of carcinogenetic activity. Needle-like crystals such as asbestos have been regarded as carcinogens, but with respect to so-called needle effects, the real carcinogens could be substances that have weak or no detectable carcinogenetic effects and are not regarded as carcinogens under ordinary conditions (in the absence of needles) because they themselves cannot pass through barriers to reach their targets (the nuclear DNA), and needle-like crystals are just helping weak carcinogens reach their targets and intensifying their carcinogenic activities. These possibilities should be examined in the future.

## Materials and methods

### Insects and plants

Newly hatched larvae of a line of the Eri silkmoth, *Samia ricini*, (Saturniidae), maintained at our institute as experimental insects were used for bioassays of the defensive effects of raphides and its synergism with bromelain. The Eri silkmoth larvae have been successfully used in bioassays to evaluate plant defense levels and to detect novel plant defense factors against herbivorous insects, such as the effects of leaf-rolling [33], defensive effects of cysteine proteases [15], detection of sugar-mimic alkaloids [34] and novel chitin-binding defense protein MLX56 [35] from mulberry latex, and detection of the novel defense protein BPLP from phloem exudate of cucurbit plants [36]. The castor oil plant, *Ricinus communi*s (Euphorbiaceae), maintained at our institute as a natural host plant of the Eri silkworm, was used in the bioassays. Young plant individuals that were sown 2 to 3 months prior to the bioassays were used in bioassays.

### Collection and purification of raphides from kiwifruits and observation of the purified raphide using an electron microscope

For the bioassays, it was necessary to collect natural raphides and purify them. We chose kiwifruits as a source of raphides because kiwifruits are commercially available, and softer and less fibrous than other commercially available sources of raphides such as taro tubers and pineapple fruits. Commercially obtained kiwifruits (cultivar Hayward) were cut transversely and the inner pericarp area adjacent to the seeds (referred to as the locular region), where idioblasts specialized in containing raphides concentrate [5], was collected. One gram of locular tissue was placed in each microtube (1.5 ml), 200 μl of 4 M CaCl2 solution was added, and then the tissue was gently homogenized using a pellet mixer (1.5 ml; Treff Lab, Switzerland). The homogenate was centrifuged (15,000 g, 10 min) and the supernatant was discarded. Then the pellet was resuspended in 1 ml of heavy liquid (6.35 M CsCl, 0.4 M CaCl2, 1.8 g/cm3). The suspension was then centrifuged (15,000 g, 10 min). Raphides, which were heavier than the heavy liquid (>2 g/cm3), sedimented and formed a pellet, while fruit pulp, which was lighter than the heavy liquid (ca. 1.4 g/cm^3^) but which still contains raphides, formed floating matter. The floating matter was then mixed with the supernatant in the microtube using the pellet mixer, taking special care not to disturb the sedimented pellet of raphides. Then it was centrifuged again (15,000 g, 10 min). Additional raphides that were separated from the pulp increased the amount of the pellet consisting of raphides. This cycle was repeated 4 times. Then, the supernatant and floating matter consisting of pulp were discarded and the pellet of raphides was collected and washed with 0.04 M CaCl2 solution. After drying and measuring the mass of collected raphides, raphides were finally kept as a suspension in 0.04 M CaCl2 solution at a concentration of 37.5 μg/μl. By this method, 8.3 mg of raphides was purified from 100 g of the locular region of kiwifruit. In order to check whether the purified raphides retained an intact needle-like shape, the purified raphides were observed using an electron microscope as follows. The purified raphides were suspended in 0.0004 M CaCl2 solution at a concentration of 37.5 μg/μl, and this suspension were dropped on adhesive tape attached on the metal block platform. After drying in air, the raphides were coated with gold, and were observed using a model JSM-6301F scanning electron microscope (JEOL, Tokyo, Japan). The SEM images of the purified raphides indicated that the purified raphides retained an intact sharp needle-like shape with a length around 0.1 mm or 100 μm (but without grooves and barb-like shapes observed in raphides of Araceae plants [12]) (Figure 1).

### Preparation of raphide-free kiwifruit extract

One gram of locular tissue of commercially obtained kiwifruit (Hayward) was placed in a microtube (1.5 ml) and was homogenized using a pellet mixer (1.5 ml; Treff Lab, Switzerland). Then, the homogenate was centrifuged (15,000 g, 10 min) and the supernatant was collected. The supernatant was centrifuged again. After the third centrifugation, the supernatant was collected as raphide-free kiwifruit extract.

### Bioassays

Leaf pieces of the castor oil plant (3 cm×3 cm, ca. 0.1 g) used for a single series of bioassay (∼6 pieces) were cut from a single young palmate leaf that had just reached its mature size (1 piece from each lobe). These leaf pieces were painted with 100 ml of solutions containing 4 mM CaCl2, (to prevent raphides from melting), 1% glycerol (used as an agent to spread or adhere the raphides), 0 mg, 0.2 mg (low dose), 2 mg or 4 mg of bromelain (B-4882, lyophilized powder purified from pineapple stem, 3.6 unit/mg (this protease unit was defined by Sigma using Na-Z-L-lysine p-nitrophenyl ester as a substrate, and is different from the unit that was defined by us using casein as a substrate and are used elsewhere in the present study); Sigma), 0% or 40% of raphide-free kiwifruit extract, 0 μg, 46.9 μg (low dose), 375 μg or 750 μg of raphides purified from kiwifruit, and 0 μg or 375 μg of amorphous calcium oxalate crystals (Wako Pure Chemical Industries, Ltd., Osaka, Japan). The solutions were painted on the underside of the leaf piece and smeared evenly on the surface using yellow pipette tips. After the leaf surface dried, neonate Eri silkmoth larvae were placed on the leaf surface and allowed to the leaf pieces for 1 day, and then the larval mass and mortality were measured.

### Protease assays and definition of unit

Kiwifruits (cultivar Hayward) that were harvested in late fall were cut, placed in a microtube (1.5 ml), and crushed (homogenized) using a pellet mixer (1.5 ml; Treff Lab, Switzerland) on the day of harvest. The homogenate was centrifuged (15,000 g, 10 min) and the supernatant was collected for protease assays. One hundred μl of sodium phosphate buffer (50 mM, pH 7.0) containing supernatants or enzymes were mixed with 1 ml of reaction solution containing 50 mM sodium phosphate (pH 7.0), 5 mM cysteine, 1 mM EDTA, and 1% casein as a substrate. Reactions were performed at 25°C for 30 min, then 1 ml of 20% trichloroacetate was added to terminate the reactions. After centrifugation (10,000 g, 10 min), supernatants were collected, then the absorptions at 280 nm (A280, 1 cm light path) were analyzed. One unit was defined as the enzyme activity that made a 0.001 increase in A280 per minute at the reaction condition described above.
